# Incidence and Risk Factors for Low Anterior Resection Syndrome following Trans-Anal Total Mesorectal Excision

**DOI:** 10.3390/jcm13020437

**Published:** 2024-01-13

**Authors:** Shani Y. Parnasa, Ido Mizrahi, Brigitte Helou, Adiel Cohen, Mahmoud Abu Gazala, Alon J. Pikarsky, Noam Shussman

**Affiliations:** Department of General Surgery, Hadassah Medical Organization and Faculty of Medicine, Hebrew University of Jerusalem, Jerusalem 91120, Israel; shani.parnasa@mail.huji.ac.il (S.Y.P.);

**Keywords:** proctectomy, rectal cancer, low anterior resection syndrome, trans-anal total mesorectal excision

## Abstract

Background: Trans-anal total mesorectal excision (Ta-TME) is a novel approach for the resection of rectal cancer. Low anterior resection syndrome (LARS) is a frequent functional disorder that might follow restorative proctectomy. Data regarding bowel function after Ta-TME are scarce. The aim of this study was to evaluate the incidence and risk factors for the development of LARS following Ta-TME. Methods: A prospectively maintained database of all patients who underwent Ta-TME for rectal cancer at our institution was reviewed. All patients who were operated on from January 2018 to December 2021 were evaluated. The LARS score questionnaire was used via telephone interviews. Incidence, severity and risk factors for LARS were evaluated. Results: Eighty-five patients underwent Ta-TME for rectal cancer between January 2018 and December 2021. Thirty-five patients were excluded due to ostomy status, death, local disease recurrence, ileal pouch or lack of compliance. Fifty patients were included in the analysis. LARS was diagnosed in 76% of patients. Anastomosis distance from dentate line was identified as a risk factor for LARS via multivariate analysis (*p* = 0.042). Neo-adjuvant therapy, hand sewn anastomosis and anastomotic leak did not increase the risk of LARS. Conclusion: LARS is a frequent condition following ta-TME, as it is used for other approaches to low anterior resection. Anastomosis distance from dentate line is an independent risk factor for LARS. In this study neo-adjuvant therapy, hand sewn anastomosis and anastomotic leak did not increase the risk of LARS. Further studies with longer follow-up times are required to better understand the functional outcomes following Ta-TME.

## 1. Introduction

The adoption of total mesorectal excision (TME) as a standard of care and the evolution of neo-adjuvant chemo-radiotherapy, as well as adjuvant treatment protocols, in the last few decades have improved the local control of patients suffering from rectal cancer [1,2]. Minimally invasive approaches are gaining popularity due to shorter hospital stays, less pain, reduced risk of perioperative morbidity and faster recovery compared to open surgery [3,4]. However, laparoscopic TME has significant technical difficulties, especially in patients with a narrow male pelvis, obesity and bulky tumors. This raises concerns regarding longer operative time, increased risk of anastomotic leakage, incomplete tumor resection due to distal or circumferential resection margin positivity and local tumor recurrence [5,6]. In 2009, trans-anal total mesorectal excision (Ta-TME) was proposed to overcome these technical limitations [7]. Ta-TME was found to be a feasible and safe approach that enhances the visualization of the surgical planes in the mid and low pelvis, potentially allowing for a more accurate dissection compared to trans-abdominal TME and potentially decreasing the rate of conversion to open surgery, as well as rates of circumferential and distal resection margin positivity [7,8,9,10,11].

Up to 80% of rectal cancer patients experience a range of evacuation difficulties following surgery, known as low anterior resection syndrome (LARS), with up to 50% of these patients in various series experiencing severe symptoms (major LARS) with impaired quality of life [12,13,14].

There are concerns that Ta-TME might result in worse LARS compared to trans-abdominal TME. Firstly, the distance of anastomosis from the anus in Ta-TME is potentially shorter compared to trans-abdominal TME [15]. Secondly, the dilatation of the anal canal during Ta-TME may result in damage to the anal sphincter muscles. Finally, dissection in the lower pelvis, especially during the learning curve, might injure the pelvic floor muscles innervation. Nevertheless, these concerns are not supported by data [15]. A systematic review of the literature suggested no significant differences in functional outcomes following Ta-TME compared to trans-abdominal TME [16].

The purpose of this study was to evaluate incidence and risk factors for LARS in patients undergoing Ta-TME for rectal cancer.

## 2. Materials and Methods

### 2.1. Study Design and Patient Inclusion

This study was conducted under the supervision of the Hadassah Medical Center institutional review board (approval number 0413-19-HMO). Consecutive patients who underwent Ta-TME for rectal cancer at our institution between January 2018 and December 2021 were included in a prospectively maintained colorectal cancer database and evaluated for inclusion in this study. The exclusion criteria included the following: patients in whom a permanent colostomy rather than an anastomosis was performed (very low Hartmann’s procedure due to the patient’s request due to fear of low quality of life), patients in whom the closure of the diverting stoma was not carried out; patients with local tumor recurrence, patients with impaired cognitive function or language difficulty who could not complete the questionnaire and patients who were unable or unwilling to participate. All participating patients provided informed consent prior to their inclusion in this study.

### 2.2. Perioperative Management and Surgical Technique

Surgery was performed 4–12 weeks after the completion of neoadjuvant treatment in patients who required it. All patients received a mechanical and antibiotic bowel preparation the day before surgery and received prophylactic intravenous antibiotics at induction. All Ta-TME procedures were performed in a synchronous two-team approach by four fellowship-trained colon and rectal surgeons (IM, MAG, AJP and NS). A medial-to-lateral approach was utilized laparoscopically, with high-ligation of the inferior mesenteric artery, division of the inferior mesenteric vein at the inferior border of the pancreas, full mobilization of the splenic flexure and entrance to the TME planes from above. Synchronously, the trans-anal team placed a purse-string suture distal to the tumor. A full-thickness proctotomy was performed distal to the purse-string, and the TME dissection was carried out bottom-up until rendezvous with the abdominal team’s dissection in the pelvis. The specimen was retrieved through the anus. The anastomosis to the rectum was either performed side to end with a circumferential mechanical stapler or hand-sewn in cases where it was impossible to use a stapler due to short distance from the anal canal. A Jackson–Pratt drain was left next to the anastomosis and was taken out once drainage was completely clear prior to discharge from the hospital. A diverting loop ileostomy was created routinely.

Stoma reversal was performed at least 3 months postoperatively and 1 month after the completion of adjuvant therapy when indicated, occurring after evaluating the anastomosis with a digital rectal examination, as well as a water-soluble contrast enema.

Patient-reported outcome measures

**Figure 1:**
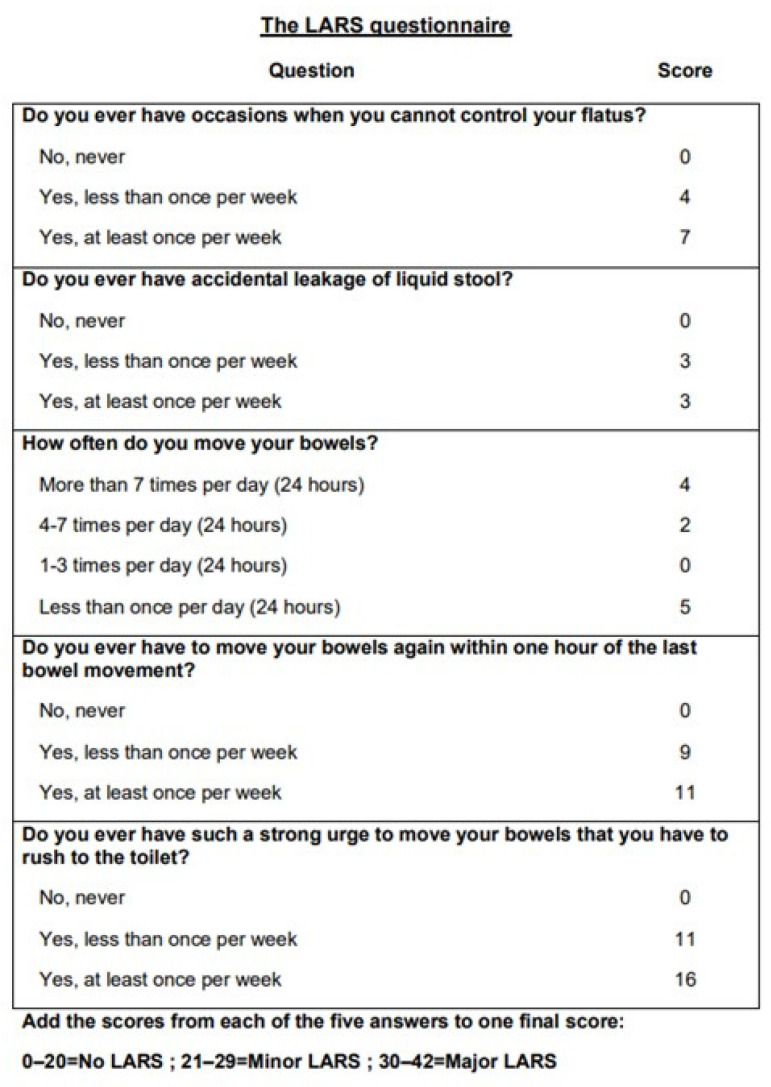
The LARS questionnaire.

Patients who met the inclusion criteria were approached by a surgeon member of the research team who did not take part in the surgery or in the perioperative care (SYP, BH or AC) via a telephone call. The patient was asked to answer the LARS questionnaire, which is a validated tool for scoring bowel dysfunction after low anterior resection for colorectal cancer. It includes five questions regarding the prevalence of flatus or liquid stool incontinence, bowel frequency and the clustering of stools and urgency. Based on the total score, LARS is categorized as nonexistent (score 0–20), minor (score 21–29) or major (score 30–42) (Figure 1) [17,18].

### 2.3. Baseline Data

Patient characteristics, including gender, age, body mass index (BMI), and neoadjuvant treatment, were collected. Operative and postoperative data included type and the distance of the anastomosis from the anus, the creation of diverting ileostomy or colostomy, the length of hospital stay, and operative and postoperative complications. Postoperative data, including adjuvant chemotherapy, time to stoma closure, time to local recurrence (defined as tumor recurrence next to the anastomosis) and time to distant recurrence (defined as metastasis in other sites), were also obtained from patients’ charts.

### 2.4. Study Outcomes

The primary outcomes were defined as the prevalence and severity of LARS. Secondary outcomes included the identification of risk factors associated with LARS.

### 2.5. Statistical Analysis

Results are presented as the median and interquartile range (IQR) for continuous variables and as frequencies for categorical variables. Spearman’s correlation was used to evaluate the relationship between LARS score and tumors, as well as measure the anastomosis distance from the dentate line. Univariate analyses, as well as the Kruskal–Wallis and Chi-square tests, were used for comparisons between patients with no LARS, minor LARS and major LARS. Comparisons between patients with and without LARS symptoms were performed via the Mann–Whitney and χ^2^ tests as appropriate. A multiple logistic regression was used to investigate the relationship between LARS symptoms and a set of potential risk factors. The set of independent variables that were inserted into the multivariate model was found to be at the <0.1 level of significance in the above-mentioned univariate analyses. The level of significance used for all analyses was two-tailed and set at *p* < 0.05. The SPSS statistical package (Version 28, SSPS Inc., Chicago, IL, USA) was used for all statistical analyses.

## 3. Results

Between January 2018 and December 2021, 85 patients underwent Ta-TME for rectal cancer at our institution. Thirty-one patients were excluded from the analysis: 22 patients still had their diverting ileostomy or had a permanent colostomy, 1 patient underwent a restorative proctocolectomy with ileal pouch-anal anastomosis, 3 patients developed local recurrence and 5 patients died.

**Figure 2:**
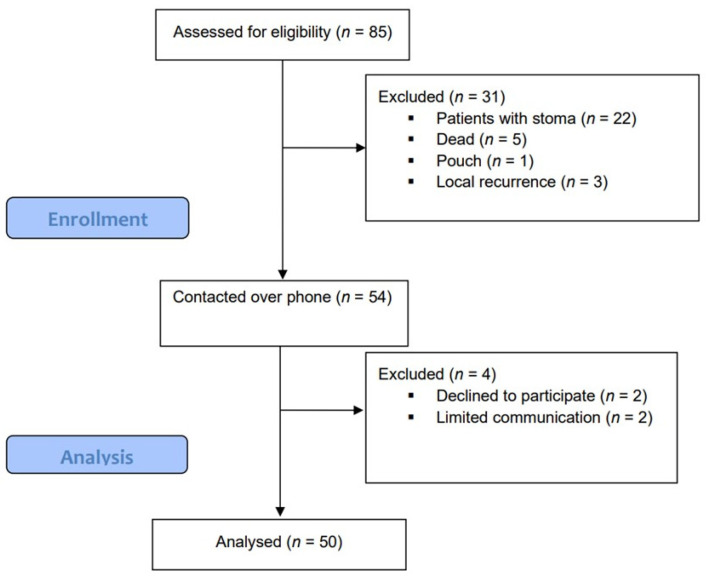
CONSORT diagram.

Fifty-four patients were eligible for inclusion, of whom four were excluded after communicating with them; two patients declined to participate, and with two other patients, there was limited communication. The response rate was 92.6%. Figure 2 shows the Consolidated Standards of Reporting Trials (CONSORT) diagram.

Among the 50 patients included in the study, the majority (31 patients, 62.0%) were male. The median age was 58.0 years (IQR 49.3–66.3), and the median body mass index was 27.1 kg/m2 (IQR 24.7–29.6). Forty-five patients (90.0%) were categorized as American Society of Anesthesiologists (ASA) class 1 or 2. The distribution of the tumors’ stage is described in Table 1.

In total, 41 patients (82%) received neo-adjuvant treatment, 34 patients (68%) received long-course chemoradiotherapy (28 radiation fractions with doses of 1.8 Gray per fraction, administered five days a week for 5.5 weeks up to a total dose of 50.4 Gray with concurrent administration of oral Capacitebine), 4 patients (8%) received short-course radiotherapy alone (five radiation fractions with doses of 5 Gray per fraction, administered in five daily fractions over the course of 5 consecutive days up to a total dose of 25 Gray) and 3 patients (6%) received short-course radiotherapy followed by systemic chemotherapy (four courses of 5-Fluorouracil with Oxaliplatin, “total neo-adjuvant therapy”–TNT). None of the patients received adjuvant radiotherapy—all the radiation treatments were given preoperatively. Four patients underwent Ta-TME as a completion following local excision (trans-anal excision or endoscopic submucosal dissection) of a primary tumor that was found to be more advanced than expected on the pathological examination of the local excision specimen.

The median operative time was 3.2 h (IQR 2.8–3.8). All patients included in this study underwent a primary anastomosis with a diverting ileostomy, as mentioned above. Anastomosis was performed with a circular stapler in 30 patients (60%) or hand-sewn in 20 patients (40%). The median tumor distance from the dentate line was 5 cm (IQR 3–7), and the median anastomosis distance from the dentate line was 3 cm (IQR 1–3.75).

Intraoperative complications included bleeding, requiring blood transfusion in two patients (4%) and conversion to open surgery in three patients (6%). Three patients (6%) developed anastomotic leakage postoperatively, two of whom presented with fever and one with purulent drainage via the pelvic drain left next to the anastomosis at the time of surgery. None of these patients required reoperation or CT-guided drainage. The median hospital stay was 8 days (IQR 7–12) (Table 1).

The evaluation of LARS was performed at a median follow-up time of seven months following stoma reversal. A total of 38 patients (76%) developed LARS following the takedown of their ileostomy. The median LARS score was 32 (IQR 21.5–38). Of the 38 patients with LARS, 6 (12%) had Minor LARS, and 32 (64%) had Major LARS (Table 2).

Shorter tumor distance, and as a result, shorter anastomosis distance from the dentate line were not associated with higher LARS scores for univariate analysis (Table 2), but for multivariate analysis, both were associated with higher LARS scores (*p* = 0.034 and *p* = 0.042, respectively). No association was found between age, gender, BMI, smoking, ASA score, preoperative stage or operative time and LARS score. Intraoperative and postoperative adverse events including anastomotic leakage did not affect the incidence or the severity of LARS, nor did neo-adjuvant or adjuvant treatment (Table 2). Moreover, the type of neo-adjuvant therapy had no significant effect on Major LARS (64.7% long-course chemo-radiotherapy versus 57.1% short-course radiotherapy, *p* = 0.50).

Protective loop ileostomies were reversed at a median of 6 months (IQR 3–7) after the index surgery (Table 1). The time from index surgery to stoma reversal had no impact on LARS scores (Table 2). Median follow-up after stoma reversal was 7 months (IQR 5–11 months) (Table 1), and there was no association between shorter follow-up and worse LARS (Table 2).

The prevalence of Major LARS was similar in patients who had a hand-sewn anastomosis and patients who had a stapled anastomosis (68.4% versus 65.5%, respectively). Among patients who underwent a stapled anastomosis, the stapler’s diameter (29 mm versus 33 mm) did not affect the incidence or severity of LARS.

Five patients who were included in the analysis later underwent an enterostomy: two patients underwent it due to severe LARS, one patient underwent it due to a benign anastomotic stenosis, and two patients underwent it due to local tumor recurrence.

## 4. Discussion

This study describes LARS scores of a cohort of the first fifty patients following restorative Ta-TME for rectal cancer at our institution. In our study, 64% of the patients reported major LARS at a median follow-up time of seven months following stoma reversal. This percentage of major LARS is within the expected range following trans-abdominal TME, which is reported to range between 25% and 80%, depending on time from surgery, anastomosis distance from the anus and neo-adjuvant radiotherapy [18,19,20,21]. Our study also showed increased risk of LARS as the anastomosis distance from the dentate line was shorter. Nevertheless, we failed to demonstrate any association of preoperative radiation, type of preoperative treatment protocol or time from closure of the ileostomy with the presence or severity of LARS.

Kneist et al. were the first to report LARS after Ta-TME for low rectal cancer in ten patients [22]. They reported that only one of their patients (10%) experienced major LARS six months following stoma reversal. However, they reported a median LARS score of 28 (range 9–38) at three months after surgery and 26 (range 9–32) at six months after surgery. Pontallier et al., on the other hand, reported major LARS 12 months after stoma closure in 82% of patients with coloanal anastomosis after Ta-TME [23]. Thus, the prevalence of major LARS in 64% of the patients in our cohort is acceptable and expected due to the relatively short distance of the anastomoses from the dentate line and the relatively short median follow-up time. A debate exists regarding the improvement of LARS over time following the restoration of bowel continuity following proctectomy. According to a recent literature review, LARS symptoms are more severe and occur more frequently during the first four months following stoma closure but improve and stabilize between the first and second year after surgery [24]. Nevertheless, this improvement over time is not a consensus, and other studies reported prevalence of LARS as high as 47.5% after a follow-up period of 13.7 years [17,23].

Other factors have been found to significantly affect the incidence of LARS. These include neo-adjuvant radiotherapy, the creation of a defunctioning ileostomy and extending the time for ileostomy reversal beyond six months [25,26]. However, these factors did not have a negative impact on LARS symptoms in our series. Previous studies point out anastomotic leakage as a predisposing factor for LARS following TME [17,27]. Our cohort included three patients with a history of anastomotic leakage, which did not increase the prevalence or the severity of LARS.

Our cohort included a relatively high number of patients with hand-sewn anastomoses (40%). This high rate reflects the nature of Ta-TME, which is kept for patients with mid and low rectal tumors seeking sphincter-preserving surgery. A hand-sewn anastomosis was previously found to be a risk factor for worse functional short-term outcomes after rectal resection [14]. Even though hand sewn anastomoses by nature have a shorter distance to dentate line, and even though in our cohort a low anastomosis was indeed a risk factor for LARS, surprisingly, for multivariate analysis, we found no correlation between anastomosis type (stapled versus hand-sewn) and worse functional outcomes. Keller et al. reported that longer trans-anal operative times were associated with worse LARS scores, which may be attributed to anal sphincter damage caused by endo-anal instrumentation during the trans-anal approach [28]. We found no correlation between major LARS and overall operative time.

This study has several limitations. Firstly, the nature of a single-arm, non-comparative retrospective study of 50 patients limits its ability to draw meaningful conclusions with respect to factors associated with LARS. This limitation is inherent to our series of rectal cancer patients since patients undergoing elective surgery for mid or low rectal cancer at our institution during the inclusion period were routinely referred for Ta-TME. Another limitation is the lack of objective assessment of functional outcomes using electromyography or anorectal manometry, which could be useful in the diagnosis of damage to reservoir function, neural function, and sphincter muscle function.

## 5. Conclusions

LARS is a major consequence of any surgical approach to rectal cancer and should be well recognized by clinicians. The incidence of LARS following Ta-TME seems to be similar to that of trans-abdominal TME. Prospective controlled studies with longer follow-up times are required to further understand the functional outcomes following Ta-TME.

## Figures and Tables

**Table 1:** Demographic, preoperative, operative and postoperative characteristics.

Factor	Total Population (*n* = 50)
**Age, years, median (IQR)**	58.0 (49.3–66.3)
**BMI, kg/m^2^, median (IQR)**	27.1 (24.7–29.6)
**Sex, *n* (%)**	
Male	31 (62.0%)
Female	19 (38.0%)
**ASA score, *n* (%)**	
1	10 (20.0%)
2	35 (70.0%)
3	5 (10.0%)
**Smoking, *n* (%)**	17 (34.0%)
**Previous surgery, *n* (%)**	
TAH	3 (6.0%)
Bariatric	1 (2.0%)
Anorectal	2 (4.0%)
**Tumor height from DL, cm, median (IQR)**	5.0 (3.0–7.0)
**Preoperative staging, *n* (%)**	
1	6 (12.0%)
2	11 (22.0%)
3	31 (62.0%)
4	1 (2.0%)
Other (NET)	1 (2.0%)
**Neo-adjuvant therapy, *n* (%)**	
Short-course radiotherapy	7 (14.0%)
Long-course chemoradiotherapy	34 (68.0%)
**Anastomosis distance from DL, cm, median (IQR)**	3.0 (1.0–3.8)
**Type of anastomosis, *n* (%)**	
Stapled	30 (60.0%)
Hand-sewn	20 (40.0%)
**Operative time, hours, median (IQR)**	3.2(2.8–3.8)
**Concomitant procedures, *n* (%)**	
Right colectomy	2 (4.0%)
TAH/Salpingo-oophorectomy	1 (2.0%)
Wedge gastrectomy	1 (2.0%)
**Intraoperative complication, *n* (%)**	
Conversion to open surgery	3 (6.0%)
Hemorrhage	2 (4.0%)
**Postoperative complications, *n* (%)**	
Anastomosis leakage	3 (6.0%)
Other	23 (46.0%)
**Postoperative stay, days, median (IQR)**	8.0 (7.0–12.0)
**Adjuvant chemotherapy, *n* (%)**	31 (62.0%)
**Time to stoma reversal, months, median (IQR)**	6.0 (3.0–7.0)
**Follow-up time after stoma reversal, months, median (IQR)**	7.0 (5.0–11.0)

(IQR, interquartile range; BMI, body mass index; ASA, American Society of Anesthesiologists; TAH, total abdominal hysterectomy; DL, dentate line; NET, neuro-endocrine tumor).

**Table 2:** Comparison between LARS score groups.

	No LARS *n* = 12 (24%)	Minor LARS *n* = 6 (12%)	Major LARS *n* = 32 (64%)	*p*-Value
**Age, years, median (IQR)**	48.5 (41.3–76.8)	61.5 (54.0–71.3)	58.5 (52.0–65.8)	0.431
**Sex, *n* (%)**				0.388
Male	6 (50.0%)	5 (83.3%)	20 (62.5%)	
Female	6 (50.0%)	1 (16.7%)	12 (37.5%)	
**BMI, kg/m^2^, median (IQR)**	26.7 (24.3–29.1)	31.9 (30.2–33.2)	27.0 (24.4–29.1)	0.053
**Previous surgery, *n* (%)**				
TAH	2 (16.7%)	0 (0.0)	1 (3.1%)	0.195
Bariatric	0 (0.0)	0 (0.0)	1 (3.1%)	0.762
Anorectal	1 (8.3%)	0 (0.0)	1 (3.1%)	0.637
**Current smoking, *n* (%)**	4 (33.3%)	4 (66.7%)	9 (29.0%)	0.208
**ASA, *n* (%)**				0.935
1	2 (16.6%)	1 (16.7%)	7 (21.9%)	
2	9 (75.0%)	4 (66.7%)	22 (68.8%)	
3	1 (8.3%)	1 (16.7%)	3 (9.4%)	
**Preoperative staging, *n* (%)**				0.800
1	0 (0.0)	2 (33.3%)	4 (12.5%)	
2	4 (33.3%)	0 (0.0)	7 (22.9%)	
3	8 (66.7%)	4 (66.7%)	19 (59.4%)	
4	0 (0.0)	0 (0.0)	1 (3.1%)	
Other (NET)	0 (0.0)	0 (0.0)	1 (3.1%)	
**Neoadjuvant therapy, *n* (%)**				0.501
None	1 (9.1%)	2 (33.3%)	6 (18.8%)	
Short-course radiotherapy	3 (25.0%)	0 (0.0)	4 (12.5%)	
Long-course chemoradiotherapy	8 (66.7%)	4 (66.7%)	22 (68.8%)	
**Median tumor height from DL, cm (IQR)**	6.5 (4.3–7.0)	6.0 (4.5–8.5)	5.0 (3.0–6.0)	0.190
**Median anastomosis height from DL, cm (IQR)**	3.0 (1.0–4.8)	3.5 (2.3–5.3)	1.0 (0.0–3.0)	0.081
**Type of anastomosis, *n* (%)**				0.969
Stapled	7 (58.3%)	4 (66.7%)	19 (59.4%)	
Hand-sewn	5 (41.7%)	2 (33.3%)	13 (40.6%)	
**Concomitant procedures, *n* (%)**				0.268
Right colectomy	2 (16.6%)	0 (0.0)	0 (0.0)	
TAH/Salpingo-oophorectomy	0 (0.0)	0 (0.0)	1 (3.1%)	
Wedge gastrectomy	0 (0.0)	0 (0.0)	1 (3.1%)	
**Intraoperative complications, *n* (%)**				
Conversion to open surgery	1 (8.3%)	0 (0.0)	2 (6.3%)	0.778
Hemorrhage	1 (8.3%)	0 (0.0)	1 (3.1%)	0.637
**Operative time, hours,** **median (IQR)**	3.1 (2.8–3.6)	3.6 (2.9–4.25)	31 (2.8–4.1)	0.560
**Postoperative complications, *n* (%)**				
Anastomosis leakage	1 (8.3%)	1 (16.6%)	1 (3.1%)	0.408
Other complications	7 (58.3%)	1 (16.6%)	15 (46.9%)	0.244
**Adjuvant chemotherapy, *n* (%)**	9 (75.0%)	5 (83.3%)	17 (53.1%)	0.253
**Time to stoma reversal, months, median (IQR)**	6.5 (5.3–8.8)	5.0 (2.8–9.5)	5.0 (3.0–7.0)	0.147
**Follow-up time after stoma reversal, months, median (IQR)**	8.0 (5.0–9.0)	13.0 (6.0–21.0)	6.5 (3.8–11.0)	0.322

(LARS, low anterior resection syndrome; IQR, interquartile range; BMI, body mass index; DL, dentate line; TAH, total abdominal hysterectomy; ASA, American society of anesthesiologists score; NET, neuro-endocrine tumor).

## Data Availability

Data is unavailable publicly due to privacy and ethical restrictions.
